# Pharmacy deserts and COVID-19 risk at the census tract level in the State of Washington

**DOI:** 10.1016/j.jvacx.2022.100227

**Published:** 2022-10-17

**Authors:** Rachel Wittenauer, Parth D. Shah, Jennifer L. Bacci, Andy Stergachis

**Affiliations:** a School of Pharmacy, CHOICE Institute, University of Washington. 1956 NE Pacific St H362, Seattle, WA 98195, USA; b Hutchinson Institute for Cancer Outcomes Research (HICOR), Fred Hutch. 1100 Fairview Ave N, Seattle, WA 98109, USA; c Department of Global Health, School of Public Health, University of Washington. Hans Rosling Center, 3980 15th Ave NE, Seattle, WA 98105, USA

**Keywords:** Vaccine distribution, Pharmacy, Vaccine access, Pandemic preparedness

## Abstract

Community pharmacies are a crucial component of healthcare infrastructure, including for COVID-19 pandemic prevention services like testing and vaccination. Communities that are “pharmacy deserts,” experience healthcare inequities. However, little research has characterized where these communities are, making it difficult for local leaders to prioritize resources for them. This study identifies pharmacy deserts at the census tract level in Washington state for the first time and explores their association with COVID-19 risk. Out of 1,441 tracts, 127 were pharmacy deserts, comprising approximately 454,000 adults, or 8% of the state’s adult population. Among those tracts identified as pharmacy deserts, 67% were considered high risk for COVID-19. Solutions are needed to expand equitable access to pharmacy services in these communities. The methods and data presented herein provide healthcare leaders with information to address this pharmacy access gap in Washington and could be similarly applied to other settings. Three categories of policy changes could address health inequities found in our study: 1) improve financial incentives for pharmacists to practice in underserved areas, 2) prevent pharmacy closures, and 3) deploy innovative care delivery methods such as telehealth services.

## Highlights

• Pharmacy desert communities have worse access to key services like vaccination.
• In Washington State, over 450,000 adults (8% of adults) live in pharmacy deserts.
• Those living in pharmacy deserts were also associated with higher risk for COVID-19.
• Several policy solutions are needed to address the access gap for pharmacy services.

## Introduction

The COVID-19 pandemic has revealed inequities in access to healthcare in the United States (US), and the importance of establishing a robust healthcare and public health preparedness system. Community pharmacies are an integral component of the US healthcare infrastructure and are the most widely dispersed healthcare access point in the US. Nationally, about 89 % of adults live within 5 miles of a pharmacy [1]. Pharmacies provide a wide range of patient care services, such as vaccinations, point-of-care testing, and dispensing of medications, including public health emergency countermeasures. To standardize variation in state laws and ease national vaccine implementation during COVID-19 pandemic, the Department of Health and Human Services (HHS) issued nationwide guidance granting pharmacists, student pharmacists, and pharmacy technicians the authority to order and administer COVID-19 vaccinations [2], [3]. Leveraging community pharmacies as immunization access points relieves care burden for other healthcare providers and increases access to preventive care services for medically underserved populations [4]. As with the 2009 H1N1 influenza A pandemic response, community pharmacists are again playing key roles as immunizers on the front lines of a public health emergency [5], [6].

Despite pharmacy’s importance in patient care services and better geographic distribution compared to other healthcare provider types, many individuals still face barriers to accessing pharmacies and pharmacist-provided services depending on where they live. Communities which are both low-income and have low geographic access to pharmacies are known as “pharmacy deserts” [7]. Pharmacy deserts have seen increased attention and study during the COVID-19 pandemic because of their implications for poor access to testing and vaccinations [4], [8], [9]. In addition to COVID-19 related services, pharmacy deserts have important health implications in that proximity to pharmacies and pharmacy closures have been shown to negatively impact adherence to medications for chronic illnesses and receipt of influenza vaccination among breast cancer patients [10], [11], [12]. Further, not all communities are impacted equally by pharmacy closures, as a higher proportion of pharmacy closures are in non-white, lower-income neighborhoods [10].

Evidence is growing on the locations and characteristics of pharmacy deserts and pharmacy access in the US. These communities have been identified at the census tract or ZIP code level in certain geographies including Los Angeles County, Chicago metro area, the state of Pennsylvania, New York City, Shelby County Tennessee, in the thirty largest metropolitan areas of the United States, and at the county level for selected rural counties [7], [13], [14], [15], [16], [17], [18], [19]. These studies have also identified several social determinants of health and demographic characteristics associated with the prevalence of pharmacy deserts, including denser population, higher proportion of renters, more residents that speak English as a second language, more residents living under the federal poverty level, higher proportion of Black and Hispanic residents, and fewer health professionals who serve the area [14], [17], [18].

Gaps remain in the literature of both the geography and characteristics of pharmacy deserts. Pharmacy deserts have only been identified at the census tract level for few geographic regions. Further, no analyses have examined the relationship between pharmacy deserts and COVID-19 risk, which is important for efficient allocation of limited resources to response and recovery from COVID-19 and future pandemics. The objectives of this study were to identify communities designated as pharmacy deserts in Washington state (WA) and examine the relationship between pharmacy deserts and community COVID-19 risk. By achieving these objectives, we provide information that enables prioritization of resources for communities with greater risks and inequities associated with poor pharmacy access.

## Methods

### Data collection and sources

*Pharmacy data source.* Data were collected from four existing publicly available sources. The names and addresses of pharmacies with active licenses during 2020 were provided by the Washington State Pharmacy Association. We identified a total of 1,877 active pharmacy licenses. We then excluded pharmacies that were: 1) healthcare entities, or organizations that provide healthcare services in a setting that is not otherwise licensed by the state to purchase and possess legend drugs such as outpatient surgery centers and residential treatment facilities; 2) hospital pharmacy-associated clinics, or an individual practitioner's office; or 3) hospital pharmacies. After these exclusions, a total of 1,330 community pharmacies were included in our analytic sample. These addresses were geocoded for analysis using the GoogleMaps application programming interface (API).

*Census data source.* Data were obtained at the census tract-level for population characteristics of median income, percent of individuals living below the federal poverty level, vehicle ownership, age, and population estimates from the 2019 5-year American Community Survey (ACS) [20]. Rural-Urban Commuter Area (RUCA) codes from the Washington State Department of Health (WADOH) were used to classify each tract as urban or rural [21].

*COVID-19 risk score data source.* COVID-19 vulnerability was defined using the COVID-19 Community Vulnerability Index (CCVI). The CCVI is based on the widely-used US Centers for Disease Control and Prevention’s Social Vulnerability Index (SVI), which is based on 4 themes: socioeconomic status, household composition and disability, minority status and language, and housing type & transportation [22], [23], [24]. The CCVI then adds two more COVID-19 specific risk themes to those 4: 5) epidemiological factors such as population density and prevalence of diabetes and 6) healthcare system factors such as health system capacity and AHRQ Prevention Quality Indicator Overall Composite Score, for a total of 6 risk areas by census tract and then organized into quintiles [23]. The CDC is relying on the SVI for vaccine planning nationally, but the CCVI was chosen for this analysis because it aligned with WADOH planning efforts and includes timely COVID-19 specific measures of risk. The use of COVID-19 case data rather than a risk index in this analysis was 1) not possible due to lack of case count data availability at the census tract level, and 2) subject to limitations of its own such as the highly varied phases of the pandemic and wide distribution of vaccines in non-pharmacy settings. Instead, the CCVI provides a uniform measure of community risk which, while not directly tied to COVID-19 case counts, allows us to identify gaps between anticipated community health needs and available pharmacy services.

There are 1,454 census tracts in Washington, of which we were able to analyze 1,441 (99.1 %). Thirteen tracts were excluded because they either had a population of zero or were suppressed for having too few residents to publicly report a median income. Results were organized by local health jurisdictions (LHJs), which are the local-level health authorities in the state. Washington has 31 county health departments, three multi-county health districts and two city-county health departments, which are collectively referred to as LHJs [25].

### Outcome measures

This analysis had two outcome measures of interest to define the location of underserved communities. As our primary outcome, we identified the prevalence of pharmacy deserts at the tract level and examined which of those tracts were also at high COVID-19 risk. Second, we identified the expected capacity of pharmacies located in each census tract, as defined by the number of adults per pharmacy residing in each community.

### Pharmacy deserts

A pharmacy desert is defined as a community (census tract) which is both “low-income” and has “low access” geographically to pharmacies. This definition is widely used in other pharmacy deserts studies [7], [26], [27] and is based on the Centers for Disease Control and Prevention’s and US Department of Agriculture (USDA)’s definition of a food desert, which is a similar concept in terms of neighborhood access to resources for health [28], [29]. This analysis applied the same definitions for these variables as described by Qato et al [7]. Specifically, “low-income” is defined as tracts where the median household income is less than 80 % of the income of the nearest metropolitan area, or where more than 20 % of residents have a household income below the federal poverty line. “Low access” is defined as where more than one-third of a census tract’s population is living farther than 1 mile from the nearest pharmacy (1 mile for urban tracts, 10 miles for rural tracts, and 0.5 miles for low-vehicle-access tracts). A low vehicle access tract is one where more than 100 households do not own a vehicle. This proportion was calculated by identifying if the centroid of a census block group was located within the specified radius of a pharmacy, and if so, then considering the population of that block to be “near” a pharmacy, then dividing the population near a pharmacy over the total population of that census tract. With this approach, we were able to define populations based on proximity to pharmacies even if a specific pharmacy was not within the border of the census tract. Urban and rural status was defined using RUCA codes from WADOH [21]. Prevalence of pharmacy deserts in urban versus rural census tracts were also examined.

### Pharmacies per capita

In addition to pharmacy deserts, as a secondary outcome we characterized the expected catchment population of each pharmacy, which we defined as pharmacies per adult population per census tract. The threshold for a federally-designated Health Professional Shortage Area (HPSA) is a population to provider ratio of 3,500 or more. We used this threshold to identify areas in which pharmacies are serving a catchment area with a larger population than 3,500 people [30]. There are more pharmacy locations than primary care locations in the US (supporting a smaller catchment ratio), though patients visit pharmacies more frequently than their primary care physicians [31] (supporting a larger catchment ratio), thus the 1 per 3,500 ratio is potentially not as applicable to pharmacies as it is for primary care. However, an average catchment area size for pharmacies has yet to be defined and broadly adopted in pharmacy access literature, and so 1 per 3,500 is used for comparison purposes.

### Statistical analyses

We identified pharmacy deserts geographically and visually displayed them on a choropleth map along with COVID-19 risk. We used chi-squared tests to evaluate statistically significant differences in the 1) proportion of adults and 2) population living in pharmacy deserts by LHJ. We assessed statistical significance of mean number of pharmacies per tract in each LHJ using univariate logistic regression. We reported frequencies of pharmacy deserts by risk status in the CCVI. All analyses were performed using R Studio.

## Results

### Pharmacy deserts

Out of the 1,441 census tracts analyzed, 127 tracts were identified as pharmacy deserts. Approximately 454,000 adults lived in pharmacy deserts, or about 8 % of Washington State’s adult population. The LHJs with the highest proportion of adults living in pharmacy deserts were Okanogan County Public Health (42 %), Klickitat County Public Health (21 %), and Benton-Franklin Health District (12 %). The LHJs with the highest number of adults living in pharmacy deserts were Seattle and King County Public Health (153,711 adults), Tacoma-Pierce County Health Department (66,844 adults), and Snohomish Health District (36,339 adults). These LHJs are the health authorities of the three most populous counties in Washington State, all surrounding the Seattle-Tacoma metropolitan area. Thirteen of the 35 LHJs had no adults living in pharmacy deserts. The variation between LHJs of number of adults and proportion of adults living in pharmacy deserts was not statistically significant (*p* = 0.28). These results are summarized in Exhibit 1.

**Exhibit 1:** Adult population living in pharmacy deserts by local health jurisdiction.

	**Adults Living in Pharmacy Deserts**^1^	**Percent of Adults Living in Pharmacy Deserts**^2^	**Proportion of All Tracts in LHJ which are Pharmacy Deserts**
**Local Health Jurisdiction**			
King	153,711	8.80 %	9.57 %
Pierce	66,844	9.99 %	11.70 %
Snohomish	36,339	5.89 %	6.71 %
Spokane	26,728	6.80 %	6.67 %
Benton-Franklin	24,186	11.71 %	12.50 %
Clark	23,079	6.44 %	6.73 %
Kitsap	19,146	9.20 %	7.55 %
Thurston	17,587	8.02 %	8.16 %
Yakima	15,350	8.75 %	11.11 %
Whatcom	14,288	8.05 %	8.82 %
Okanogan	13,360	41.46 %	40.00 %
Skagit	10,200	10.41 %	10.34 %
Cowlitz	8,109	9.85 %	16.67 %
Northeast Tri County	5,720	10.96 %	15.00 %
Chelan-Douglas	4,547	5.10 %	4.55 %
Lewis	4,438	7.24 %	10.00 %
Klickitat	3,732	21.33 %	33.33 %
Grant	2,557	3.80 %	6.25 %
Asotin	1,925	10.79 %	16.67 %
Clallam	1,049	1.68 %	4.76 %
Grays Harbor	632	1.09 %	6.25 %
Mason	515	1.00 %	7.14 %
Other^3^	0	0.00 %	0.00 %

1 Not statistically significant between census tracts based on chi squared test: p = 0.28.

2 Not statistically significant between census tracts based on chi squared test: p = 0.28.

3 Other LHJs which had zero adult population living in pharmacy deserts were: Adams, Columbia, Garfield, Island, Jefferson, Kittitas, Lincoln, Pacific, San Juan, Skamania, Wahkiakum, Walla Walla, and Whitman.

About one-third (30 %; 432/1441) of all census tracts in Washington were considered “high” or “very high” risk for COVID-19 based on the CCVI. However, 67 % (85/127) of census tracts identified as pharmacy deserts in Washington were “high” or “very high” risk for COVID-19. Exhibit 2 depicts the pharmacy deserts by tract as orange for tracts that are pharmacy deserts and red for tracts that are both pharmacy deserts and “high” or “very high” COVID-19 risk. Location of pharmacies are noted as points in black on the map, and county boundaries are noted with thicker border lines than for tracts. Exhibit 3 provides the same map for King County, the most populous county in the state. An interactive HTML version of the map with additional data available is provided as Supplemental Material.

**Exhibit 2:**
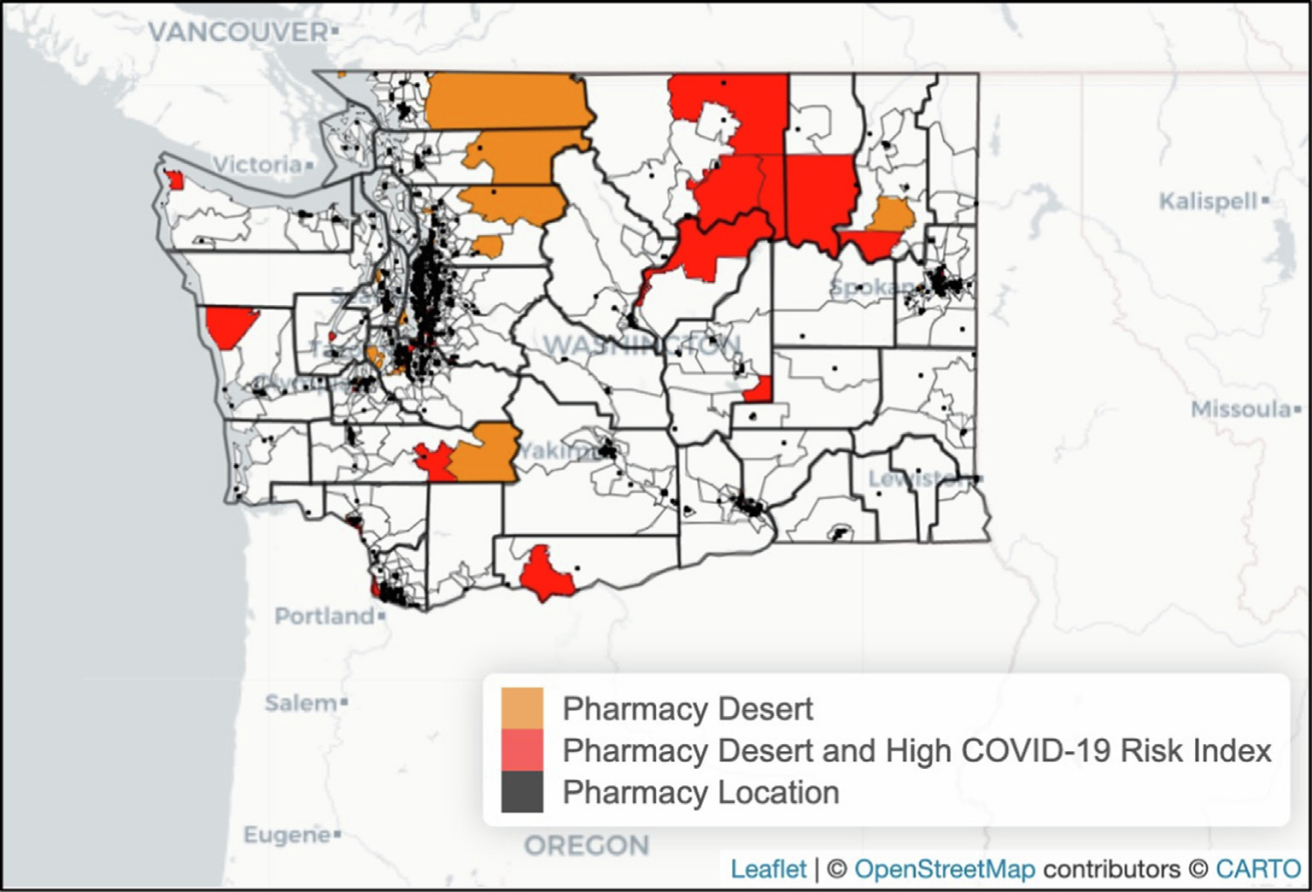
Pharmacy desert communities in Washington state.

**Exhibit 3:**
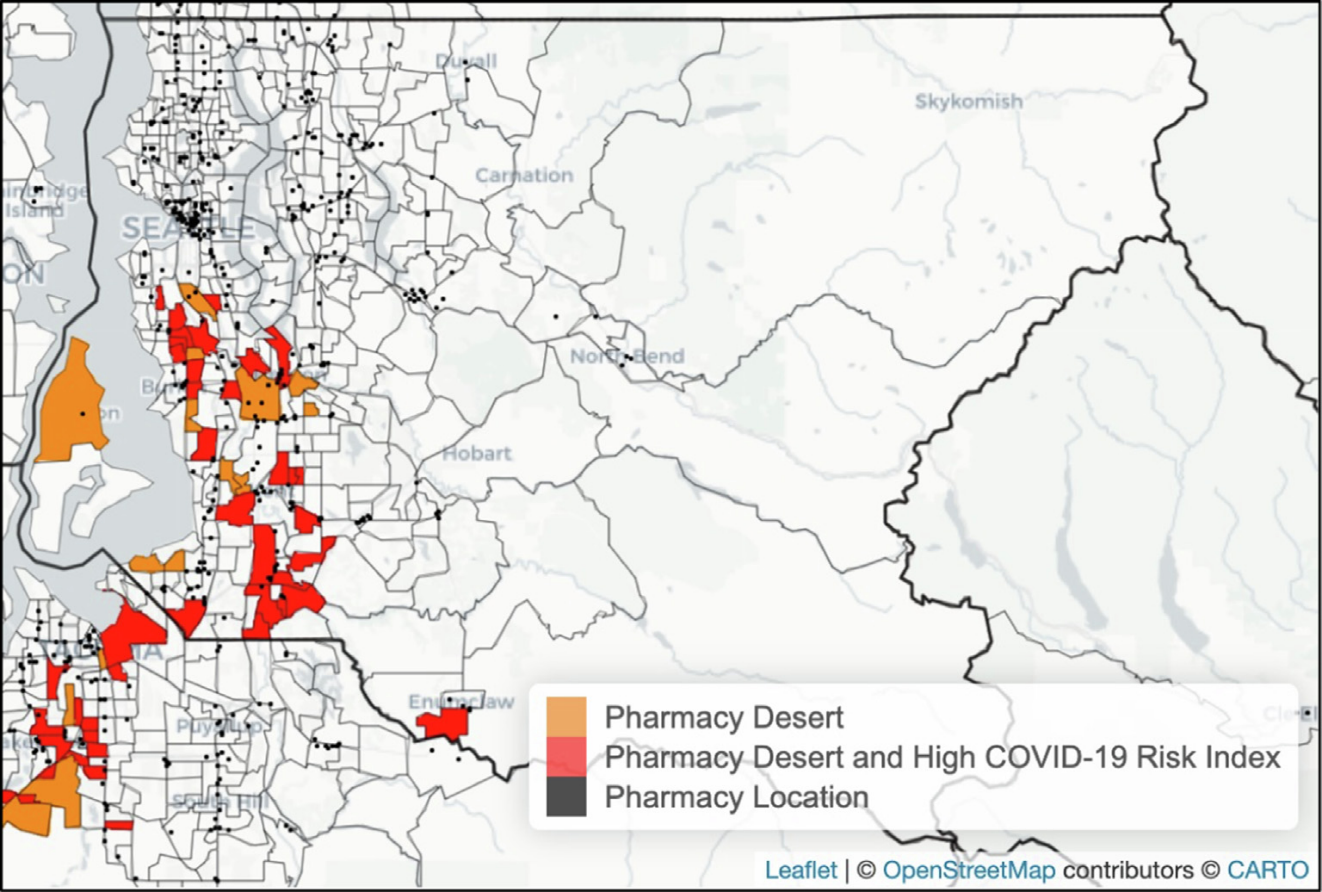
Pharmacy desert communities in King County, Washington.

A slightly higher proportion of urban communities (9 %; 111/1232) were pharmacy deserts compared to rural (8 %; 16/208), though this difference was not statistically significant (*p* = 0.52). There were 38,678 adults living in rural pharmacy deserts and 415,364 adults living in urban pharmacy deserts. There were 111 urban pharmacy deserts out of 1232 urban tracts and 16 rural pharmacy deserts out of 209 rural tracts.

### Pharmacies per capita

In examining pharmacies per adult population in Washington, 69 % (988/1441) of communities were considered healthcare shortage areas that had fewer than 1 pharmacy per 3,500 population. The mean number of pharmacies per 3,500 population in each county ranged from 0.37 in Skamania County to 2.0 in Garfield County. Of the 36 LHJs, 28 have a mean number of pharmacies per capita of less than 1 per 3,500 adult population. These data specific to LHJ are summarized in Exhibit 4.

**Exhibit 4:** Pharmacies per population by local health jurisdiction.

**Local Health Jurisdiction**	**Pharmacies per 3500 Population**	**Proportion of Adult Population Living in HPSA areas**
Skamania	0.37	83.0 %
Klickitat	0.40	100.0 %
Island	0.47	84.1 %
Clark	0.66	71.9 %
Mason	0.68	68.1 %
Walla Walla	0.70	70.2 %
San Juan	0.72	63.1 %
Whatcom	0.73	63.1 %
Skagit	0.75	74.5 %
Pierce	0.76	70.4 %
Jefferson	0.77	67.9 %
Kitsap	0.77	71.3 %
Thurston	0.80	78.4 %
King	0.81	71.2 %
Snohomish	0.81	67.2 %
Benton-Franklin	0.81	69.6 %
Chelan-Douglas	0.82	74.0 %
Adams	0.83	56.2 %
Whitman	0.84	76.9 %
Clallam	0.84	69.9 %
Cowlitz	0.85	67.2 %
Lewis	0.86	67.5 %
Yakima	0.89	61.0 %
Grant	0.89	59.0 %
Kittitas	0.92	67.8 %
Northeast Tri County	0.94	75.5 %
Wahkiakum	0.98	100.0 %
Spokane	0.99	61.4 %
Grays Harbor	1.03	50.8 %
Columbia	1.09	0.0 %
Pacific	1.16	50.1 %
Okanogan	1.20	34.5 %
Lincoln	1.26	52.9 %
Asotin	1.37	68.3 %
Garfield	2.07	0.0 %
Note: HPSA (Health Provider Shortage Area) is defined as an area with less than one provider per 3,500 population.		

## Discussion

To our knowledge, this is the first study to define pharmacy deserts at the local level in Washington and to explore the relationship between community COVID-19 risk and pharmacy deserts. Most adults who live in pharmacy deserts are in King, Pierce, and Snohomish counties, the most populous counties in Washington State. These results also indicate that communities which have poor access to pharmacies are also often those at higher risk for COVID-19. The census tract-level granularity of these results provides state and local health departments with information about opportunities to work with community pharmacies to address outbreak prevention and mitigation, as well as where the state should mobilize additional healthcare resources for communities with the greatest inequities and COVID-19 risk. Longer-term, these data could provide information for pharmacies about their community’s health needs and opportunities to expand patient care services to increase healthcare equity.

### Policy implications

Solutions or healthcare policy changes are needed to improve access to pharmacy-based patient care services and strengthen healthcare infrastructure in specific communities, especially for future public health emergency and pandemic preparedness. Several policy changes that could support a stronger pharmacy infrastructure that addresses the health inequities found in our study have been proposed and generally fall into three categories: 1) improve reimbursements, payment models, and financial incentives for practicing pharmacists to practice in underserved areas, 2) prevent closures of pharmacies, and 3) deploy innovative care delivery methods such as telehealth and mail-based services.

*Policy solution 1: Expand pharmacist reimbursement.* In April of 2021, the Pharmacy and Medically Underserved Areas Enhancement Act (H.R. 2759/S. 1362) was introduced to US Congress [32]. This act would grant pharmacists “provider status” and allow them to bill Medicare Part B for services that they regularly provide in underserved communities including medication management, point-of-care testing, immunizations, and chronic disease management [33]. Since 1987, the Centers for Medicare and Medicaid (CMS) has offered bonuses to healthcare providers in designated shortage areas, and these incentives were further expanded by the Affordable Care Act in 2011 with the goal to increase the supply of primary care services in specific underserved geographies [34], [35]. These policies were evaluated by the Department of Health and Human Services in 2015, which found that the financial incentives did indeed increase the number of providers in HPSAs, albeit only by a small amount (increase from mean of 6 to mean of 7 providers in HPSAs) [36]. Pharmacy deserts, by definition, are in low-income communities, and this analysis highlights that these communities are potentially also at higher risk for COVID-19. These communities are where gaps in access to care are the starkest and addressing these gaps will require financial support for pharmacies to feasibly provide services there. In 2015, Washington became the first state to grant pharmacists status as health care providers, including coupling that status with mandatory reimbursement for those essential health services [37]. This law allows patients to access covered medical benefits when provided in pharmacies, however the magnitude of the impact of this policy on patient access to services is yet to be fully evaluated and implementation barriers remain [37], [38], [39]. While Washington was the first state to enact a law granting pharmacists “provider status,” the Pharmacy and Medically Underserved Areas Enhancement Act still has important implications for Washington pharmacies because it would extend that “provider status” to cover Medicare services, which must be accomplished at the federal level. In states with broad pharmacist scope-of-practice regulations, such as Washington, pharmacies are not able to implement those services broadly while they remain unlinked with payments. A recent analysis by Murphy et al similarly concluded that “aligning the pharmacist business model to be comparable to other health care professionals will ensure patients receive access to pharmacist-provided cognitive patient care services.” [40] Granting pharmacists “provider status” nationally may alleviate some financial pressures in pharmacy deserts, however, until implemented, the extent of the impact of improved CMS reimbursements on pharmacy budgets remains to be seen.

*Policy solution 2: Prevent closures of community pharmacies.* A recent study found that 1 in 8 pharmacies closed between 2009 and 2015 nationally, with closures being disproportionately of independent pharmacies and of pharmacies in low-income communities [10]. Further, in November 2021, CVS, the second-largest pharmacy chain in the US, announced the upcoming closure of 900 of their community pharmacies over the next three years, which is approximately 1 in 10 of their stores [41], [42]. This is consistent with findings from a wide-ranging body literature on the nature of spatial distribution of health and community services; as one analysis stated: “the reasons behind pharmacy siting decisions are rooted primarily in market forces and are not based on need[s] of the community” [16]. Our results in this analysis found that out of the 36 local health jurisdictions in Washington, 28 of them have a mean number of pharmacies per capita that is less than one per 3,500 (the threshold definition of a Health Professional Shortage Area), and these pharmacy shortages occurred in both rural and urban areas of the state. In January 2022, House Bill 1813, “Concerning Freedom of Pharmacy Choice” was introduced to the Washington State Legislature, with the goal of expanding patient access to pharmacies not in their health insurance networks, including explicitly in pharmacy desert communities [43]. While improving CMS reimbursement and easing patient access restrictions may help alleviate some of the financial pressure on pharmacies, it will likely take additional policy, financial, or market-based incentives to keep brick-and-mortar pharmacies open, particularly in underserved communities.

*Policy solution 3: Innovate on healthcare service delivery.* In communities where local pharmacies have closed, pandemic preparedness and response services which might normally be based within a brick-and-mortar pharmacy are no longer available. Solutions to improve access to pharmacy services could also include innovations in care delivery through improved use of technology. For example, in the rural town of Shoshone, Idaho, that state’s first telehealth pharmacy opened which has sustained operations by using technology to reduce staffing costs while still providing pharmacist consultations for every prescription virtually and enabling residents to get their pharmacy needs without driving to a further city [44]. The sole remaining pharmacy in St. Charles Iowa, a town of 1,000 people, has adopted a similar model [45]. In Washington, the top three local health jurisdictions by proportion of population living in pharmacy deserts (Okanogan, Klickitat, and Benton-Franklin) are all highly rural communities, and potentially stand to benefit from innovative healthcare delivery models such as telehealth. This type of innovative model could have great potential for improving access to pharmacy-based services during a pandemic in other rural areas of the United States as well.

### Strengths and limitations

Our study has notable strengths. Rather than calculating population within reach of a pharmacy by using only centroids of tracts, this analysis used block groups, which is the most granular level of geographic detail available in the ACS. This analysis also was able to capture populations living within reach of a pharmacy even if that pharmacy was in a neighboring tract, thus presenting a more accurate picture of the proportion of population in each tract is within reach of any pharmacy. This is also the first study to define pharmacy deserts at this level of detail in Washington, and the first study of any pharmacy deserts to examine the relationship of pharmacy deserts with associated COVID-19 risk.

We also note limitations to our study. We used linear distances to calculate geographic distance to pharmacies rather than travel time or road distance, and as such the number of people living in pharmacy deserts in this analysis is likely underestimated. The population of census block groups within each tract are assumed to be homogeneously distributed for the areal interpolation calculations of population within a certain radius of a pharmacy, which does not account for the existence of parks, non-residential buildings, and other spaces where people do not reside. In terms of data inputs, the 2019 ACS survey data are estimates of the population rather than actual counts, as opposed to the 2020 Decennial census data which are not yet available but could provide further precision to these population estimates. There are multiple limitations to the CCVI used in this analysis: 1) Though it is based on the widely-used SVI, since the CCVI itself is new since the start of the pandemic, it has not been as thoroughly validated as a measure of population-level health risk. However, the social and biological components underlying the COVID-19 measures of the CCVI have substantial evidence supporting them and the CCVI has been used by WADOH for other response planning efforts; 2) The risk indices underpinning the CCVI, namely the socioeconomic status sub-component, and the parameters comprising the pharmacy desert definition are highly correlated, which limits the validity of statistical inference about the relationship between the two variables. Due to lack of data availability in ACS and the CCVI, this analysis was unable to be conducted from the lens of the 29 sovereign tribal nations in Washington. Though WADOH and LHJs collaborate government-to-government with tribal officials for COVID-19 programs, the tribal nations have separate authorities and community health programs which were not in the scope of this analysis, though populations living in these tribal areas are included in the tract and county-level lens presented in our main analysis. Lastly, while there is limited generalizability of these results outside of Washington, these methods and publicly-available data can be straightforwardly applied to other states throughout the US.

While this analysis examines population-level access to pharmacies and risk with COVID-19, it should be noted that geographic access to pharmacies is not entirely synonymous with access to pharmacy-based services. Many prescription medications are delivered by mail, consultations can occur virtually through telemedicine, and COVID-19 testing and vaccination services are available at a variety of community and medical settings beyond pharmacies. While examining characteristics of specific pharmacy services or pharmacy operational characteristics (e.g., staffing) was not within the scope of this study, future studies could examine these components to further contextualize the relationship between pharmacy access and disease risk to obtain more precise estimates of gaps in care coverage during public health crises when healthcare resources are strained.

## Conclusions

There are approximately 450,000 adults living in Washington communities that have low financial resources and poor geographic access to pharmacies. These “pharmacy desert” communities are more often associated with higher community-level risk of COVID-19 than communities that are not pharmacy deserts, and the results highlighted in this analysis can help provide local health jurisdictions with actionable location-based data to fill in these gaps in access to pharmacies. Longer-term, a combination of several policies and programs to address gaps in access to vital pharmacy-based services will be an important component of future pandemic preparedness and response, and broader equity in access to healthcare both in Washington and beyond.

## Declaration of Competing Interest

The authors declare that they have no known competing financial interests or personal relationships that could have appeared to influence the work reported in this paper.

## Data Availability

Data will be made available on request.
